# A135 DEEPLY EMBEDDED ESOPHAGEAL FISHBONE REMOVED BY ENDOSCOPIC MUCOSAL DISSECTION: CASE REPORT AND LITERATURE REVIEW

**DOI:** 10.1093/jcag/gwad061.135

**Published:** 2024-02-14

**Authors:** S Jugnundan, S Gupta, C Teshima

**Affiliations:** Medicine, University of Toronto, Toronto, ON, Canada; Medicine, University of Toronto, Toronto, ON, Canada; Medicine, University of Toronto, Toronto, ON, Canada

## Abstract

**Background:**

Foreign body ingestion is frequently encountered in gastroenterology clinical practice. While most objects pass spontaneously, sharp objects, most commonly bony fragments in adults, have a tendency to become lodged in the esophagus. The European and American Society of Gastrointestinal Endoscopy both recommend emergent endoscopy for sharp objects or bones impacted in the esophagus. However, there is little guidance regarding management of deeply embedded foreign bodies, that may not be visible intra-luminally or that may have penetrated the submucosa or muscularis propria.

**Aims:**

In this case report, we describe successful endoscopic retrieval of a deeply embedded fishbone in the distal esophagus. A literature review was also conducted to evaluate the current evidence surrounding the use of endoscopic ultrasound (EUS) and submucosal dissection (ESD) to localize and remove deeply embedded foreign bodies.

**Methods:**

A literature search on EUS and ESD use in foreign body retrieval was performed via MEDLINE/PubMed (September 2023) using relevant keywords and MeSH terms.

**Results:**

CASE DESCRIPTION

A 50-year-old man presented to hospital with several hours of dysphagia after consuming fish. A fishbone deeply embedded through the submucosa into the muscularis propria was identified on CT scan and EUS. ESD was employed to free the bone and a fully covered metal stent was deployed to close the defect. The patient had no immediate complications and recovered well post-procedure.

Our literature search revealed a total of 6 case reports detailing endoscopic management of penetrating foreign bodies. One case used EUS to localize an object which was subsequently removed surgically. The remaining 5 cases all used ESD to remove submucosal objects without any short or long-term complications.

**Conclusions:**

Clinical history is critical to prompt diagnosis of foreign body ingestion, and it is crucial to be familiar with the radiologic characteristics of the suspected object. Fishbones are frequently encountered and are most commonly radiolucent on plain films. CT scans should be obtained in these patients. Rarely, these objects will be found to be deeply penetrating. There are emerging case reports supporting the use of EUS in the localization of some of these objects, particularly when they cannot be seen intraluminally. Upon review of the literature, there is a paucity of reports describing their removal. This case report adds to the sparse literature demonstrating that ESD has an emerging role in the management of deeply penetrating foreign bodies. Following multidisciplinary discussion and consultation with the appropriate surgical service, ESD may prevent the need for otherwise invasive and potentially morbid surgical intervention.

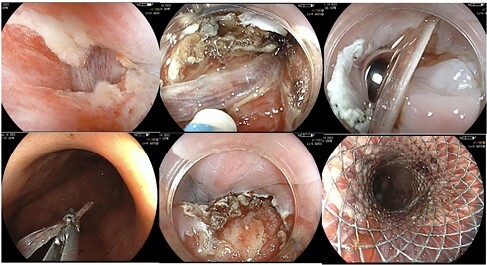

Fishbone embedded in the distal esophagus. Removed by ESD, defect closed with metal stent.

**Funding Agencies:**

None

